# Evaluation of the efficacy of *Lactobacillus plantarum* HEAL9 and *Lactobacillus paracasei* 8700:2 on aspects of common cold infections in children attending day care: a randomised, double-blind, placebo-controlled clinical study

**DOI:** 10.1007/s00394-019-02137-8

**Published:** 2019-11-16

**Authors:** Irini Lazou Ahrén, Anna Berggren, Cristina Teixeira, Titti Martinsson Niskanen, Niklas Larsson

**Affiliations:** Probi AB, Lund, Sweden

**Keywords:** Probiotic, Common cold, Respiratory tract infections, *Lactobacillus plantarum*, *Lactobacillus paracasei*, Probi Defendum^®^

## Abstract

**Background:**

The combination of *Lactobacillus plantarum* HEAL9 and *Lactobacillus paracasei* 8700:2 (commercially available as Probi Defendum^®^) has previously been reported to reduce the incidence, duration and severity of naturally acquired common colds in adults. The aim of the present study was to evaluate the impact of Probi Defendum^®^ on aspects of common cold in healthy children 1–6 years of age attending day care.

**Methods:**

A total of 131 children, out of the planned 320, were recruited into the study during 1 common cold season and randomised to consume once daily either 10^9^ CFU (colony forming units) of the probiotic product or placebo. Due to unforeseen reasons, the recruitment of more children did not continue beyond the first cold season.

**Results:**

There were 106 children that completed the study out of the 131 randomised. Daily consumption of the probiotic product for a period of 3 months significantly reduced the severity of the symptom “nasal congestion/runny nose” with a mean severity score for the whole study period of 7.5 ± 9.7 in the probiotic group and 13.9 ± 15.2 in the placebo (*p* < 0.05). Moreover, significantly less concomitant medication was used in the probiotic group. When the data were projected to a larger population corresponding to the originally estimated sample size, the results were in favour of the probiotic group regarding the reduced absence from day care (*p* < 0.05), reduced mean total severity per day in the reported episodes (*p* < 0.05) and reduced severity of the symptom “crying more than usual” (*p* < 0.05).

**Conclusion:**

Intake of Probi Defendum^®^ once daily for a period of 3 months was beneficial to children and reduced the severity of common colds.

## Introduction

Probiotics are live microorganisms which when administered in adequate amounts confer health benefits to the host [1]. Although there is an increasing interest in identifying new areas for possible health benefits from using probiotics, the two main areas for scientific evaluation remain those of gastrointestinal disorders and modulation of the immune system. The latter can be evaluated either in diseased or healthy populations using various models for studying the immune response towards for example vaccination or following an induced or community-acquired infectious disease such as the common cold. When studying the probiotic benefits at a younger age, it is common to do studies with children attending day care. This is of clinical relevance due to the easier spreading of infections among young children, in this setting, and it is also relevant due to the increased absence from day care/school and work as a result of the infections. The impact of probiotics against common upper respiratory tract infections both in adult populations and children has been reviewed and evaluated in multiple meta-analyses [2–4]. There are studies supporting the benefit with probiotic usage in respiratory tract infections and others reporting negative results. Although there is in general no doubt for the probiotic efficacy, the strain specificity is underlined as well as the importance of having strain-specific evidence for efficacy and safety, generated and confirmed in well-designed randomised clinical trials. Moreover, based on health-economic analyses, it was reported that probiotics can substantially reduce the socioeconomic burden associated with common upper respiratory tract infections experienced by both adults and children [5, 6]. The combination of *Lactobacillus plantarum* HEAL9 and *Lactobacillus paracasei* 8700:2 has previously been shown to reduce the incidence, duration and severity of common colds in adults [7, 8]. In the current study, the aim was to confirm the benefit from using the same probiotic product to reduce the incidence of upper respiratory tract infections/common colds (URTIs) in children 1–6 years of age attending day care.

## Methods

### Design of the study

The study was randomised, double-blind, placebo-controlled with the objective to evaluate the efficacy of *Lactobacillus plantarum* HEAL9 and *Lactobacillus paracasei* 8700:2 as compared to placebo, on the incidence of upper respiratory tract infections (URTIs) in children attending day care. Focus was on viral acute infections of the upper respiratory tract, i.e. common cold infections. The trial was approved by the Ethical Review Board in Uppsala, Sweden, and was registered at ClinicalTrials.gov (number NCT01935986) prior to study start. Informed consent forms signed by the caregivers were collected before randomization of the children into one of the two study groups. The recruitment was initiated in September 2013 and by the end of the common cold season 2013–2014, there were 131 children randomised out of 193 screened. The recruitment was paused during the summer months with the aim to be continued in the following season of 2014–2015. However, due to closedown of the clinical research organization (CRO), the recruitment of more study participants did not continue after the first common cold season and the total number of children randomised remained at 131.

### Sample size

Based on previous data from clinical studies with the same probiotic product in adults [7, 8], it was estimated that 40% of the subjects in the active group compared to 60% in the placebo would experience at least one URTI. The calculation of sample size was, therefore, based on an estimated difference of 20 percent-units between the probiotic group and the placebo in the incidence of URTIs. Assuming a 30% drop-out rate, an alpha level of 0.05 and a power of 80%, 160 subjects were needed in each study group. However, as mentioned above, due to unforeseen reasons, the recruitment was not completed, and the study was terminated after 131 children had been recruited.

### Study participants

Healthy children of both genders at the age of 1–6 years old, attending day care and whose caregivers had given a signed informed consent were eligible for participation in the study. Exclusion criteria were significant illness (including common cold) within the 2 weeks prior to intervention or any active systemic infection or medical condition that might require treatment or therapeutic intervention during the study, history of severe allergic reactions or anaphylaxis or any allergy to compounds of the investigational product to an extent that would jeopardize the subject or the study purpose as judged by the investigator, treatment with immune modulatory or stimulating medication or botanicals/herbal supplements (e.g. Echinacea) within 4 weeks before randomization in the study, antibiotic treatment 30 days before randomization, a history or current signs of perennial allergic rhinitis or asthma, influenza vaccination within 3 months before the start of the intervention, caregiver/caregivers smoking at home, regular consumption of probiotics as food supplements in the past 3 months before randomization, regular consumption of probiotics or probiotic fermented milk in 4 weeks prior to randomization, participation in another clinical trial during the last 4 weeks prior to the beginning of this study, incapability to comply with the study procedures, any other reason which in the opinion of the Investigator might either put the subject at risk because of participation in the study or influence the results or the subject’s ability to participate in the study.

### Study procedures

The study was advertised in the local communities and a pre-screening was done electronically. The parents answered questions related to the inclusion and exclusion criteria that determined the eligibility of their children for participation in the study. A physical screening visit was booked for collection of the signed informed consent and for confirming the compliance to the inclusion/exclusion criteria. A 2-week-long run-in period followed, during which intake of other products containing added live bacterial cultures was not allowed. The caregivers were provided with a list of the products to be avoided. They were also instructed how to daily fill in an electronic study diary about their child’s health and presence or absence from day care, starting in the last week of the run-in period and continuing until the end of the study. Caregivers were asked to daily evaluate if their child was ill or not and for every day the child was “feeling sick”, the caregivers had to fill in the additional part of the diary consisting of the validated Canadian Acute Respiratory Illness Flu Scale (CARIFS). A second study visit was scheduled at the end of the run-in period and the children were randomly assigned to consume either the probiotic product or placebo. The third and final visit at the clinic was approximately 12 weeks after the start of intervention. Adverse Events spontaneously reported by the subjects, observed or elicited based on non-leading questions by the investigator or medical personnel were collected from the time of signing the informed consent until completion of the study.

### Investigational product

The active investigational product (IP) consisted of the two probiotic bacterial strains *Lactobacillus plantarum* HEAL9 (DSM 15312) and *Lactobacillus paracasei* 8700:2 (DSM 13434). Each bacterial strain was equally represented in the total bacterial dose of 1 × 10^9^ CFU/sachet. The placebo was of identical appearance, taste and texture as the active product with the bacteria excluded. The IP was supplied in sachets containing a powder of freeze-dried bacteria and/or maltodextrin as filler. The powder was to be dissolved in 100 ml of water or other cold drink and consumed preferably at breakfast, once daily for the total length of the study (90 days). The children were randomly allocated to receive probiotic product or placebo based on a computer-generated randomization list with blocks of four. Sealed envelopes were prepared for the allocation concealment and were safely stored by the principal investigator, throughout the study. The labelling of the study product and the preparation of the sealed code envelopes was done by employees at Probi not otherwise involved in any study-related activities.

### Outcomes

The primary objective of the study was to show the benefit from using the probiotic combination as compared to placebo, on the incidence of upper respiratory infections during the intervention period of 90 days. Incidence was defined as the number of children reporting at least one infection. The secondary endpoints included the evaluation of the probiotic impact, in comparison to placebo, on the number of days with absence from day care due to URTIs as well as the duration and the severity of the documented URTIs. The assessment of both the primary and secondary endpoints was based on the information provided by the caregivers through the electronic study diary that was filled in daily for the total length of the study. The severity of the reported URTIs was evaluated by means of the validated Canadian Acute Respiratory Illness and Flu Scale (CARIFS) consisting of not only 18 items including illness-specific questions but also items related to the social behaviour and general mood of the child [9]. The start of a URTI episode was defined as the first of at least 2 days in a row with at least two symptoms scored above 0, whereas the end was defined as the last day with symptoms followed by at least 2 subsequent days with 0 score.

### Statistical analysis

Descriptive statistics (number of observations, minimum and maximum values and standard deviation or standard error of the mean when this is specified) were used and evaluated using Fisher’s exact test for the analysis of the categorical endpoints, whereas Mann–Whitney *U* test/Wilcoxon rank sum test was applied for the analysis of the continuous variables. Statistical calculations were performed using StatXact Version 10.1 and all presented *p* values are nominal, i.e. not adjusted for multiplicity. A *p* value less than 5% is considered statistically significant.

The predefined main analysis set included subjects with no major protocol deviations (as evaluated before breaking the randomization code) and a compliance of at least 80% intake of study product (PP population). However, data on the primary endpoint, incidence of common cold episodes, are also presented for the intention to treat (ITT) population. In addition, a theoretical projection of the obtained results, to the aimed number of approximately 300 participants, was applied using each participant three times. It was assumed that the difference between the groups remained the same in the population of 300 children as obtained in the population of 99. Data on safety are presented for the ITT population only.

## Results

**Fig. 1:**
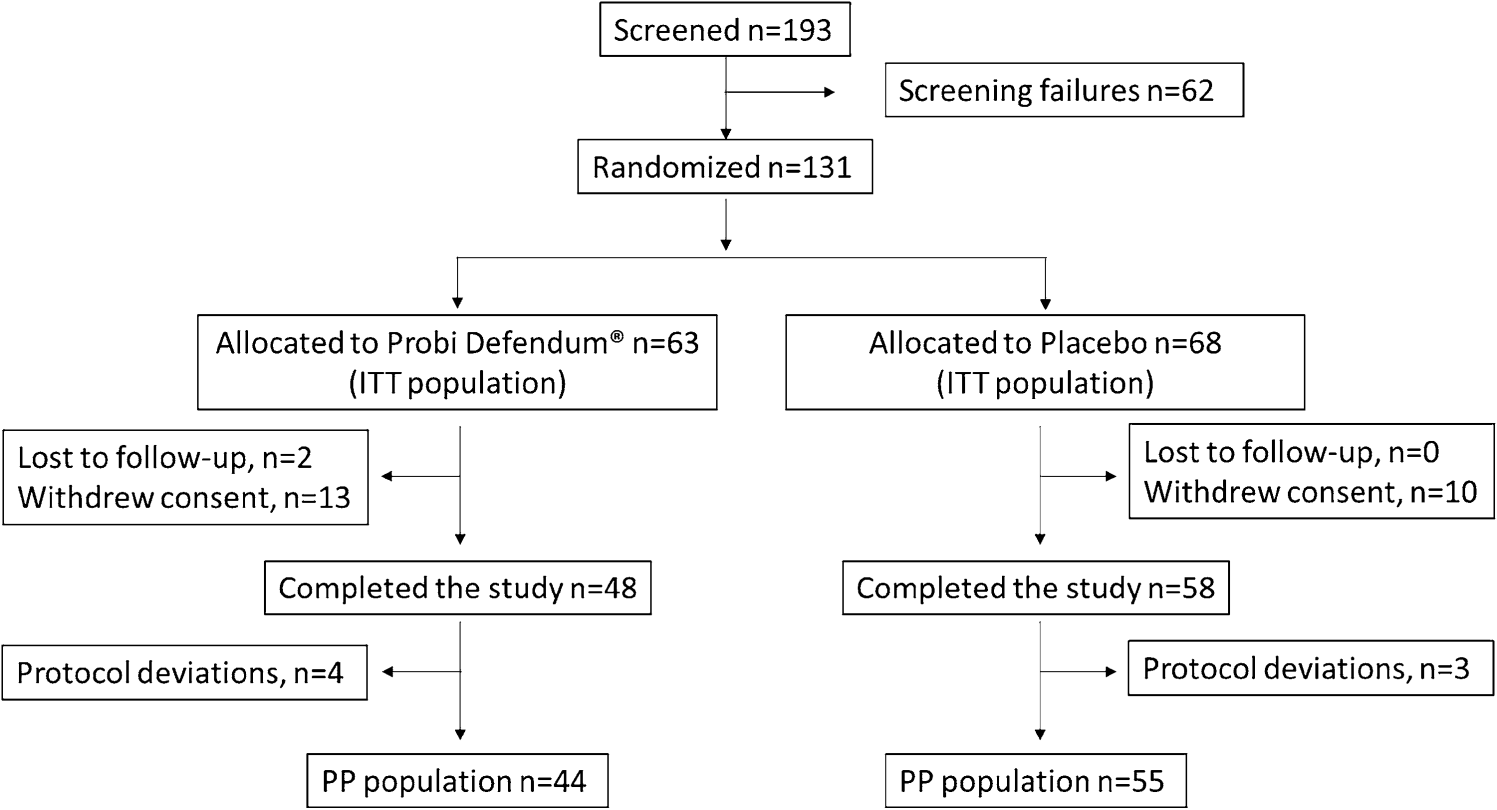
Study flowchart

As presented in Fig. 1, 131 healthy children were recruited during one common cold season and were randomly allocated to receive either probiotic product (63) or placebo (68). There were no ongoing colds at the time of recruitment. In total, 25 children discontinued the study (11.4%) mostly due to parental withdrawal of informed consent. There were 106 children who completed the study, 48 (76.2%) in the probiotic and 58 (85.3%) in the placebo group. Out of these, 7 presented with major protocol deviations and 44 in the probiotic compared to 55 in the placebo group were found to be eligible for inclusion in the PP population. The two study groups did not differ at baseline regarding gender and age (Table 1). The mean age of the children in both groups was 3.1 ± 1.4 years and the majority were younger than 5 years old.

**Table 1:** Baseline characteristics (ITT population)

	Total	Probi Defendum^®^	Placebo
Children randomised, *n* (%)	131 (100)	63 (48.1)	68 (51.9)
Males, *n* (%)	75 (57.2)	32 (50.8)	43 (63.2)
Females, *n* (%)	56 (42.7)	31 (49.2)	25 (36.8)
Age in years. mean (SD)	3.1 ± 1.4	3.1 ± 1.4	3.1 ± 1.4
1–2 years old, *n* (%)	48 (36.6)	24 (38.1)	24 (35.3)
3–4 years old, *n* (%)	54 (41.2)	24 (38.1)	29 (42.6)
5–6 years old, *n* (%)	30 (22.9)	15 (23.8)	15 (22.1)

### Incidence of URTIs

A total of 131 URTIs were reported by 99 children in the PP population but there was no statistical difference between the groups with regards to the primary endpoint, incidence of URTIs (Table 2). In the probiotic group, 34 out of 44 children (77.3%) reported 69 URTIs in total compared to 44 out of 55 children (78.2%) in the placebo group reporting 62 infections. Furthermore, there were no differences between the treatment groups regarding the incidence of common colds neither in the ITT population nor in the age subgroups. Although the study focused on viral acute infections of the upper respiratory tract, there were three children in the probiotic group and four children in the placebo with reported usage of antibiotics because of symptoms in the upper airways.

**Table 2:** Number of children with URTIs in the PP and ITT populations, *n* (%)

	Probi Defendum^®^	Placebo	*p*
PP, *n* (%)	44	55	
At least 1 episode	34 (77.3)	43 (78.2)	1
> 1 episode	22 (50.0)	23 (41.8)	NS
> 2 episodes	8 (20.4)	15 (27.3)	NS
> 3 episodes	1 (2.3)	4 (7.3)	NS
ITT, *n* (%)	63	68	
At least 1 episode	41 (65)	47 (69.1)	0.7
> 1 episode	21 (33.3)	23 (33.8)	NS
> 2 episodes	10 (15.8)	15 (22.8)	NS
> 3 episodes	1 (1.6)	4 (5.9)	NS

### Absence from day care due to sickness

Intake of the probiotic product resulted in less days with absence from day care as compared to intake of placebo with a mean of 1.7 ± 2.7 and 2.4 ± 3.5 days, respectively (*p* = 0.2). Although the difference was not statistically significant between the groups in the current study population, the projected data showed that a significance in favour of the probiotic would have been obtained in a similar population of 300 children (*p* = 0.026).

### Duration of URTIs

There was no significant difference between the treatment groups regarding the mean duration of the reported URTIs overall in the study (6.2 days in the probiotic group and 5.8 days in the placebo) or for the first and second episodes. However, the third episode was significantly shorter in the probiotic group compared to the placebo group (4.0 ± 2.2 days and 6.6 ± 2.8 days, respectively (*p* = 0.023). There were 8 children in the probiotic and 15 in the placebo group that experienced a third episode.

### Severity of URTIs

**Fig. 2:**
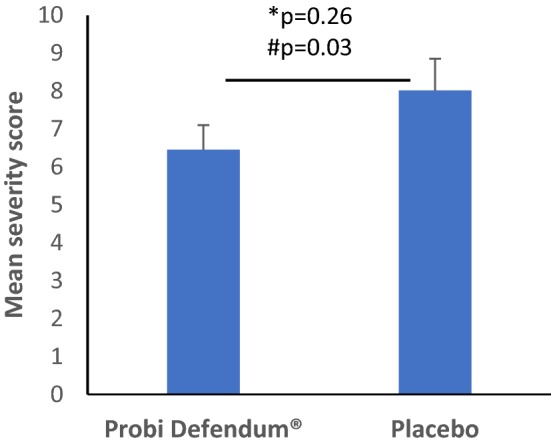
Mean total severity score per day in the episode based on CARIFS data for the subjects that had at least one URTI (SEM), *Wilcoxon rank sum test with data from the current study population, ^#^Wilcoxon rank sum test with data projected to a larger population

The validated questionnaire CARIFS was applied for measuring the severity of the reported common colds. The mean total severity measured per day in the reported episodes was lower in the probiotic group as compared to the placebo (6.5 ± 3.8 and 8.0 ± 5.5, respectively, non-significant difference) (Fig. 2). Projecting the data to the intended sample size of 300 children, the difference between the groups became significant (*p* = 0.034). Analysis of the individual items included in CARIFS revealed a significant difference between the groups, already among the 99 children included in the actual PP population, for severity of the symptom “nasal congestion/runny nose” (*p* = 0.024), as presented in Table 3. Furthermore, projecting the data showed significant reduction by the probiotic, also for the severity of the item “crying more than usual” (*p* = 0.0037). In addition, in the projected data, an almost significant difference was observed for the item “clingy” that seemed to be improved in the probiotic as compared to the placebo group (*p* = 0.051). There were no significant differences between the treatment groups for the remaining 15 items in CARIFS based on the actual or the projected data.

**Table 3:** Mean severity score for selected symptoms during the entire study, for subjects that had at least one URTI (SD)

	Probi Defendum^®^*n* = 34	Placebo *n* = 43	p*	p*^#^
CARIFS item “nasal congestion/runny nose”	7.5 (9.7)	13.9 (15.2)	0.024	0.0001
CARIFS item “crying more than usual”	1.9 (3.5)	4.0 (7.2)	0.097	0.004
CARIFS item “clingy”	3.9 (6.8)	5.4 (10.3)	0.265	0.051

*Wilcoxon rank sum test

^#^Projected data to 300 participants

### Adverse events

There was no difference between the two study groups in the incidence of AEs (Table 4). There were 35 children in the probiotic group and 32 in placebo who reported at least 1 AE. Most of the children reported only 1 AE, whereas 9 and 14 children in the probiotic and placebo group, respectively, reported more than 1 AE. As presented in Table 5, there were significantly more gastrointestinal-related AEs in the probiotic group as compared to placebo which seemed primarily to be driven by the higher number of cases with vomiting. There were eight AEs in total that were rated as possibly related to the treatment. Three of these were reported in the placebo (diarrhoea, flatulence, loose stools), whereas five were reported in the probiotic group (diarrhoea, flatulence, loose stools, stomach pain, gastroenteritis). All eight cases were considered mild or moderate.

**Table 4:** Incidence of reported adverse events (ITT population)

	Total (*n* = 106)	Probi Defendum^®^ (*n* = 63)	Placebo (*n* = 68)	p*
Subjects with at least one AE, *n* (%)	67 (51.1)	35 (55.5)	32 (47.1)	0.38
Subjects with one AE, *n* (%)	44 (33.6)	26 (41.3)	18 (26.5)	0.09
Subjects with > 1 AE, *n* (%)	23 (17.5)	9 (14.3)	14 (20.6)	0.36

*Fischer’s exact test

**Table 5:** Frequency of AEs (% of all AEs in the respective treatment group)

	Total	Probi Defendum^®^	Placebo	p*^#^
Total no. of AEs	97	46	51	
Gastrointestinal related AEs (%)	61 (62.8)	34 (73.9)	27 (52.9)	0.037
Constipation	5 (5.1)	2 (4.3)	3 (5.9)	NS
Diarrhoea	5 (5.1)	2 (4.3)	3 (5.9)	NS
Flatulence	2 (2.1)	1 (2.8)	1 (1.96)	NS
Loose stools	6 (6.2)	3 (6.5)	3 (5.9)	NS
Stomach pain	2 (2.1)	2 (4.3)	0	NS
Gastroenteritis	30 (30.9)	15 (32.6)	15 (29.4)	NS
Vomiting	11 (11.3)	9 (19.6)	2 (3.9)	0.092
Non-gastrointestinal-related AEs (%)	36 (37.1)	12 (26.1)	24 (47.0)	0.037
AEs possibly related to the treatment (%)	8 (8.4)	5 (10.9)	3 (5.9)	NS

*Fischer’s exact test

^#^Compared to the total nr of AEs reported in the same group

### Concomitant medication

As presented in Table 6, there were in total 35 subjects in the PP group who reported usage of concomitant medication, 11 subjects in the probiotic and 24 in the placebo group (*p* = 0.06). The corresponding numbers in the ITT population were 15 subjects in the probiotic group and 25 in the placebo (*p* = 0.13). Most of the children used concomitant medication due to respiratory tract infections, but there were also a few cases associated with gastrointestinal disorders, primarily constipation. Examples of other reasons for medicating included toothache, pinworm infection and skin reaction due to the paediatric disease varicella. Overall, there were 62 occasions with concomitant medication reported in the study and 48 of these were linked to participants in the placebo. As a result, the mean number of medications used in the PP population was 0.3 ± 0.65 in the probiotic group compared to 0.9 ± 1.4 in the placebo (*p* = 0.018).

**Table 6:** Number of children with concomitant medication (PP population)

	Total (*n* = 99)	Probi Defendum^®^ (*n* = 44)	Placebo (*n* = 55)	p*
Subjects with any concomitant medication, *n* (%)^a^	35 (35.3)	11 (25.0)	24 (43.6)	0.06
RTI-related, *n* (%)^b^	28 (80.0)	10 (0.9)	18 (75.0)	NS
GI-related, *n* (%)^b^	2 (5.7)	0	2 (8.3)	NS
Other reasons, *n* (%)^b^	7 (20.0)	4 (36.4)	3 (12.5)	NS

^a^Percentage of all the subjects in the corresponding group

^b^Percentage of the subjects, in the corresponding group, with concomitant medication

*Fischer’s exact test

## Discussion

The probiotic impact on common upper respiratory tract infections (common cold) has been widely evaluated in randomised clinical trials (RCTs) and summarized in multiple reviews and meta-analyses [2, 4, 10, 11]. Common cold is a viral infection occurring frequently in the community. Children can experience 6–8 colds per year, with the younger children having up to 11 episodes [12], while adults have in average 2–4 colds per year. Although the common cold is not a serious or life-threatening disease, at least not in healthy populations, it does confer a socioeconomic burden for the individuals and the health care systems. There are multiple probiotic bacteria that have been evaluated for efficacy against respiratory tract infections with some examples being *Lactobacillus rhamnosus* GG, *Lactobacillus acidophilus* NCFM and *Bifidobacterium animalis* subsp. *lactis* BB-12 [13–17]. Although there is no doubt that probiotics can reduce the burden of common colds, the strain specificity is highlighted. In addition, the age of the study population, length of intervention and even the formulation may impact the results.

The aim of the current study was to evaluate if daily intake of *Lactobacillus plantarum* HEAL9 and *Lactobacillus paracasei* 8700:2 (10^9^ CFU/day), for a period of 3 months, improved aspects of common colds in Swedish children attending day care. The study was designed to include 320 children, but the recruitment was unexpectedly stopped when 131 children had been randomised, as already described. The data collected were, therefore, analysed for the actual population in the study and for an identical projected population that was three times larger to approximate the original estimated requirement of 320 children.

Intake of *Lactobacillus plantarum* HEAL9 and *Lactobacillus paracasei* 8700:2 did not prevent the acquisition of upper respiratory tract infections in the study population nor did it reduce the mean duration of the infections compared to that of the placebo. There was, however, a significantly shorter duration of the third cold episode that was reported by 8 children in the probiotic group and 15 children in the placebo. Moreover, there was a reduced absence from day care in the current population with a mean of 1.7 days in the probiotic group compared to 2.4 days in placebo. The difference was significant following projection of the results to a larger population (*p* = 0.026).

Similar results were seen for the severity score of the reported respiratory tract infections. Mean total severity score per day in the episodes was 8.0 in the placebo compared to 6.5 in the probiotic group (*p* = 0.226), a difference that reached statistical significance in the projected data (*p* = 0.034). Interestingly, the individual CARIFS item “nasal congestion/runny nose” was significantly milder in the probiotic group within the actual study population with a mean severity score of 7.5 compared to 13.9 in the placebo (*p* = 0.024). Moreover, there were trends for reduced severity in the probiotic group also for the items “crying more than usual” and “clingy”, two items of high relevance when evaluating the severity of infections in young children who cannot express themselves verbally.

The benefit against common cold infections following intake of *Lactobacillus plantarum* HEAL9 and *Lactobacillus paracasei* 8700:2 at 10^9^ CFU/day for a period of 3 months has previously been reported in adult populations [7, 8]. Berggren et al. reported a reduced incidence of common cold infections from 67% in the control group to 55% in the probiotic group (*p* < 0.05) and a reduction in the number of days with symptoms from 8.6 days in the control to 6.2 days in the participants having consumed the probiotic [7]. There was also a significant reduction in severity of the pharyngeal symptoms following intervention with the probiotics. In the second RCT in adults evaluating the benefit from using the same probiotic, Busch et al. reported the significantly shorter cold episodes in the probiotic compared to the placebo group (5.6 and 6.7 days, respectively) and a reduced severity of the colds in the probiotic group with 9 of 13 evaluated symptoms being significantly reduced compared to the placebo [8].

Interestingly, the relative difference between the study groups for the incidence of common colds in the study by Berggren et al. was larger the more episodes the participants were experiencing [7]. This could be compared with the reduced duration of the third episode experienced by the children in the current study, despite the absence of efficacy on the incidence of the colds. Although the observation in the current study is based on a small number of children, it could reflect the ability of the specific combination of two probiotic bacteria to strengthen or prime the immune system, in a preventative manner, for a more efficient defence against upcoming infections. In the study by Rask et al., it was reported that intake of *Lactobacillus paracasei* 8700:2 tended to increase the population of natural killer (NK)-T cells (*p* = 0.06) and also to increase the expression of the memory marker CD45R0 on the surface of CD8+ lymphocytes [18]. Intake of the strain *Lactobacillus plantarum* 299v (Lp299v) significantly increased the expression of the activation marker CD25 on the surface of CD8+ T cells (*p* = 0.01). In addition, there was an increased phagocytic activity of polymorphonuclear (PMN) leukocytes and monocytes isolated from study participants who had consumed either the *Lactobacillus paracasei* (*p* = 0.05) or the *Lactobacillus plantarum* strain (*p* = 0.06) compared to placebo. Lp299v is genetically very similar to *Lactobacillus plantarum* HEAL9 (present in Probi Defendum^®^) that could hypothetically be expected to behave in a similar way to Lp299v. The innate cell-mediated immune functions induced by *L. paracasei* 8700:2 and the activation of T-lymphocytes by *L. plantarum* in the study by Rask et al. [18] could support an anti-viral activity and could possibly explain the mechanism behind the effects seen in the current study and the studies by Berggren et al. [7] and Busch et al. [8].

In the current study, there was no difference between the study groups regarding incidence and total number of adverse events reported by the children. There were, however, more gastrointestinal adverse events reported in the probiotic group compared to the placebo which seemed to be explained by the higher frequency of vomiting, a type of adverse event reported also in other studies following administration of probiotics to young children [4]. All the cases with “vomiting” were described as either “not related” or “unlikely related” to the study product.

More children reported usage of concomitant medication in the placebo group compared to the probiotic (*p* = 0.06), primarily for respiratory tract-associated symptoms. This could indirectly reflect a reduced severity of the respiratory symptoms in the probiotic group, which is in agreement with the significantly reduced severity of the nasal symptom “runny nose”. One could also consider that the higher usage of concomitant medication by the children in the placebo group resulted in milder symptoms that may have impacted the measured difference in symptom severity between the two study groups.

The current study evaluated if orally administered probiotics can prevent the acquisition of common cold infections in the natural setting and/or reduce the severity of the acquired colds. A different approach for studying the probiotic efficacy in the respiratory tract is the topical application in the nose using nasal sprays. This application has primarily been evaluated for the treatment of bacterially caused chronic rhinosinusitis [19, 20] as a means of reducing bacterial complications following a common cold. The possible combination of nasal probiotics with antibiotics has also been discussed for reducing the risk for post-antibiotic nasal colonization by Gram-negative bacteria or *Staphylococcus aureus*. Furthermore, there seems to be a nasal probiotic efficacy in allergic rhinitis with supportive evidence primarily for strains of *Lactobacillus paracasei* [21, 22]. In this latter case, probiotics are considered as a possible adjuvant therapy for the treatment of allergic rhinitis. However, both with chronic rhinosinusitis and allergic rhinitis the aim with the probiotic application has primarily been to treat or counteract the worsening of the existing symptoms which is different from the preventive application of probiotics that has been evaluated in the current study and other studies with naturally acquired or induced common colds.

The strength of this study is that although the actual sample size was lower than aimed for, there were still significant differences detected between the study groups in favour of the preventative beneficial usage of the probiotic. A weakness, on the other hand, is the unmet efficacy concerning the primary endpoint that was the incidence of common colds. However, when *Lactobacillus plantarum* HEAL9 and *Lactobacillus paracasei* 8700:2 were evaluated in adults, there was a reduced incidence in one of the studies but not in the other. As already mentioned, this discrepancy could reflect the difficulties with studying the probiotic impact on the immune system that is a complex network of many interacting parameters. Taken together, the current study provides for the first time evidence that *Lactobacillus plantarum* HEAL9 and *Lactobacillus paracasei* 8700:2 reduce the severity of common colds in children as reflected in symptom relief, reduced need for medication during the study and reduced absence from day care due to sickness.
