# Correction to: Evaluation of the efficacy of *Lactobacillus plantarum* HEAL9 and *Lactobacillus paracasei* 8700:2 on aspects of common cold infections in children attending day care: a randomised, double-blind, placebo-controlled clinical study

**DOI:** 10.1007/s00394-019-02156-5

**Published:** 2019-12-19

**Authors:** Irini Lazou Ahrén, Anna Berggren, Cristina Teixeira, Titti Martinsson Niskanen, Niklas Larsson

**Affiliations:** Probi AB, Lund, Sweden

## Correction to: European Journal of Nutrition 10.1007/s00394-019-02137-8

The original version of this article unfortunately contained a mistake. A number in Table 6 has been corrected.

The percentage should be 10 (91) instead of 10 (0.9) in the second row of the column “Probi Defendum^®^ (*n* = 44)”. The corrected Table 6 is given below.

**Table 6:** Number of children with concomitant medication (PP population)

	Total (*n* = 99)	Probi Defendum^®^ (*n* = 44)	Placebo (*n* = 55)	p*
Subjects with any concomitant medication, *n* (%)^a^	35 (35.3)	11 (25.0)	24 (43.6)	0.06
RTI-related, *n* (%)^b^	28 (80.0)	10 (91)	18 (75.0)	NS
GI-related, *n* (%)^b^	2 (5.7)	0	2 (8.3)	NS
Other reasons, *n* (%)^b^	7 (20.0)	4 (36.4)	3 (12.5)	NS

^a^Percentage of all the subjects in the corresponding group

^b^Percentage of the subjects, in the corresponding group, with concomitant medication

*Fischer’s exact test

